# Risk of Congenital Anomalies after the Opening of Landfill Sites

**DOI:** 10.1289/ehp.7487

**Published:** 2005-06-14

**Authors:** Stephen R. Palmer, Frank D.J. Dunstan, Hilary Fielder, David L. Fone, Gary Higgs, Martyn L. Senior

**Affiliations:** 1 Department of Epidemiology, Statistics and Public Health, Wales College of Medicine, Cardiff, Wales; 2 GIS Research Centre, School of Computing, University of Glamorgan, Pontypridd, Wales; 3 Department of City and Regional Planning, Cardiff University, Cardiff, Wales

**Keywords:** congenital malformations, epidemiology, landfill, small-area health statistics

## Abstract

Concern that living near a particular landfill site in Wales caused increased risk of births with congenital malformations led us to examine whether residents living close to 24 landfill sites in Wales experienced increased rates of congenital anomalies after the landfills opened compared with before they opened. We carried out a small-area study in which expected rates of congenital anomalies in births to mothers living within 2 km of the sites, before and after opening of the sites, were estimated from a logistic regression model fitted to all births in residents living at least 4 km away from these sites and hence not likely to be subject to contamination from a landfill, adjusting for hospital catchment area, year of birth, sex, maternal age, and socioeconomic deprivation score. We investigated all births from 1983 through 1997 with at least one recorded congenital anomaly [*International Classification of Diseases, Ninth Revision (*ICD-9*)*, codes 7400–7599; *International Statistical Classification of Diseases and Related Health Problems, Tenth Revision* (ICD-10), codes Q000–Q999]. The ratio of the observed to expected rates of congenital anomalies before landfills opened was 0.87 [95% confidence interval (CI), 0.75–1.00], and this increased to 1.21 (95% CI, 1.04–1.40) after opening, giving a standardized risk ratio of 1.39 (95% CI, 1.12–1.72). Enhanced congenital malformation surveillance data collected from 1998 through 2000 showed a standardized risk ratio of 1.04 (95% CI, 0.88–1.21). Causal inferences are difficult because of possible biases from incomplete case ascertainment, lack of data on individual-level exposures, and other socioeconomic and lifestyle factors that may confound a relationship with area of residence. However, the increase in risk after the sites opened requires continued enhanced surveillance of congenital anomalies, and site-specific chemical exposure studies.

The possibility of adverse health effects of living near landfills has become a major public health issue (Dolk et al. 1998; Elliott et al. 2001; Fielder et al. 2000a, 2000b). The EUROHAZ-CON study of hazardous waste landfill sites in five European countries found a 33% increase in the risk of congenital abnormalities in infants born to mothers living within 3 km of the sites (Dolk et al. 1998). The Small Area Health Statistics Unit study of more than 19,000 landfill sites in Great Britain (Elliott et al. 2001) showed a statistically significant but very small (1%) increase in congenital anomalies in babies born to women living within 2 km of any landfill site. The authors acknowledged that this U.K. national study suffered from problems in the accuracy of environmental data and from the possible bias of differential underreporting of congenital anomalies by hospitals.

Our present study arose from health concerns expressed by residents that chemicals emitted from a single landfill site in the Rhondda Valley, Wales, were the cause of congenital anomalies. These fears led to a major public outcry, direct action, a public inquiry (Purchon 2001), and an international review by the Agency for Toxic Substances and Disease Registry (2003). In preliminary epidemiologic studies we found that rates of notified congenital anomalies in the local government areas where complaints of smells occurred were significantly higher than rates in socioeconomically matched control areas, but this was the case before as well as after the landfill opened (Fielder et al. 2000b). We therefore carried out a new study to test the null hypothesis that the opening of new land-fills in Wales was not associated with increased rates of congenital anomalies in nearby residents by comparing rates before and after sites opened. Validation of environmental data parameters was undertaken with the help of local government personnel and the government-funded Environment Agency. We also adjusted for the potential bias caused by differential underreporting of congenital anomalies by different hospitals.

## Materials and Methods

### Birth data.

Cases of statutorily notifiable congenital anomalies were obtained from the U.K. Office of National Statistics for 1983 to 1997. All births with at least one recorded anomaly with *International Classification of Diseases, Ninth Revision* [ICD-9; World Health Organization (WHO) 1979] and *International Statistical Classification of Diseases and Related Health Problems, Tenth Revision* (ICD-10; WHO 1994] codes in the range of 7400–7599 for ICD-9 and Q000–Q999 for ICD-10 were included. Subgroup analysis was carried out for two major groups where numbers of cases were reasonably large, namely, chromosomal anomalies (ICD-9 codes 7580–7589, ICD-10 codes Q900–Q999) and cardiovascular defects (ICD-9 codes 7450–7479, ICD-10 codes Q210–Q289) and also for abdominal wall defects (ICD-9 codes 7567, ICD-10 codes Q79.2–Q79.3) that were of special concern in Wales (Fielder et al. 2000b).

The denominator was taken from the computerized Child Health System (Andrews et al. 1996) register of live births in Wales from 1983 through 1997, which recorded sex, gestation, and birth weight of each child and demographic details of the mother. We used a geographic information system point-in-polygon technique to assign area-based socioeconomic deprivation scores (Townsend et al. 1988) to each birth, using the score of the census enumeration district containing the postal code of maternal residence. Cases of anomalies were linked to birth records if there were an identical date of birth and at least two of the following four matching variables: birth weight, postal code, mother’s date of birth, and mother’s age. We also linked unique pairs where date of birth differed but three or more of the other four variables matched. This method resulted in matches being found for 6,780 of the 7,233 (94%) birth defects records; the remaining 453 (6%) could not be linked. Unmatched births with congenital anomalies were not more likely to reside near landfill sites. Hospital of birth was not available to us for all individual births. We therefore input hospital of birth on the basis of postal code of residence within catchment areas derived from a subset of the Child Health System database.

For the period 1998 through 2000, we obtained congenital anomalies data from the newly established Wales Congenital Anomaly Register and Information Service (CARIS) (Botting 2000) that became operational in 1998 and that has substantially increased reporting rates in Wales (Greenacre et al. 2000). Only live births were included to match to the Child Health System. Of the 2,633 congenital anomalies in live births reported, 2,534 (96%) were matched. There was no statistical association between unmatched cases and distance from landfill.

### Landfill data.

We sought information from the Environment Agency to identify new landfill sites in Wales opening from 1985 and licensed to accept commercial, industrial, and household waste; the dates on which they became operational; details of the site capacity; and types of waste accepted as described by the categories of the National Waste Classification Scheme 1999 (Environment Agency 1999). We identified landfills that were licensed to take chemical waste (types 28, 29) and those that subsequently introduced containment (removal of landfill from one part to new specially lined sections), and/or gas venting. We checked these data with the respective local government departments and made substantial corrections, especially to the dates when sites began to receive waste. The Environment Agency identified 20 sites that opened during the period, and four additional sites where there was major expansion or significant change of use during the study period.

We calculated the distance between the maternal residence at the time of birth and the grid reference of the centroid of the site as determined by the Environment Agency. Where sites were situated in proximity, we allocated the birth to the nearest site. Elliott et al. (2001) defined exposure as births within 2 km of the centroid of sites, whereas Dolk et al. (1998) used 3 km as the definition. Results were similar for the 2 km and 3 km distances; unless otherwise stated, we report only the 2 km findings.

### Statistical analysis.

Because congenital anomalies registration rates changed over the 15-year period and the ascertainment rate varied considerably between hospitals, we calculated “expected” rates from a logistic regression model fitted to the set of all births at least 4 km away from any of the major sites. This model incorporated maternal age, hospital of birth, year of birth, deprivation (quintiles of the distribution of Townsend scores), and the sex of the baby. We derived a predicted probability for each birth, and by summing these over a given area we calculated the expected number of congenital anomalies. We then compared this with the observed number to calculate a standardized risk ratio. We derived 95% confidence intervals (CIs) for the ratio (Breslow and Day 1994).

To examine the appropriateness of concentric circles to define exposure, we estimated the spatial distribution of risk by calculating observed and expected frequencies for squares with 250-m sides. To reduce the influence of random variation, we calculated standardized values (observed–expected/square root of expected) and applied a kernel smoothing method (Diggle 1990) to these as a descriptive tool to visualize risk patterns.

## Results

### 1983–1997.

Between 1983 and 1997, 542,682 births were identified on the Child Health System in Wales, of which 6,148 (1.1%) had at least one congenital anomaly within the codes defined in ICD-9 (WHO 1979) and ICD-10 (WHO 1994). This included 97 abdominal wall defects, 416 chromosomal anomalies, and 544 cardiovascular defects. Proportions of congenital anomalies did not vary significantly by maternal age or deprivation scores except for chromosomal abnormalities, where the proportion increased significantly with maternal age.

For the pooled data from the 20 sites opening during the period, the ratio of observed to expected congenital anomalies within 2 km before opening was 0.87 (95% CI, 0.75–1.00), and this increased to 1.21 (95% CI, 1.04–1.40) after opening, with a standardized risk ratio of 1.39 (95% CI, 1.12–1.72) (Table 1). In the 15 sites that introduced new containment units after opening, there was a small increase in the rate ratio, but the 95% CI included unity. Gas control was introduced in 10 sites, and in these a 25% fall in rate ratio was observed, but again, the 95% CI included unity.

Seven sites introduced both containment and gas control. In the 8 sites that introduced only containment, the rate ratio was 1.47 (95% CI, 0.45–7.56). For the three sites that introduced only gas control, the numbers of cases were too small to allow the calculation of a CI. Using a definition of exposure of 3 km, with consequent larger numbers of exposed births, the rate ratio was 3.93 (95% CI, 1.43–16.95) for containment only and 0.16 (95% CI, 0.04–0.45) for gas control only.

Six sites that opened during the study period were licensed to accept chemical waste. In these sites the rate before opening was higher than for all sites, but the standardized risk ratios after opening were very similar to the overall risk ratios. In the analysis of all pooled data for the prespecified subcategories of congenital anomalies, the risk ratio for cardiovascular defects (n = 544) within 2 km before and after opening was 1.74 (95% CI, 0.83–3.78). The risk ratios were 2.33 (95% CI, 0.53–14.0) for abdominal defects (n = 97) and 1.27 (95% CI, 0.64–2.58) for chromosomal abnormalities (n = 416).

There was substantial variation in risk ratios between sites, and the small numbers of births around many of the sites gave wide CIs. For 16 sites there were sufficient data to calculate site-specific risk ratios; in 10 sites, the point estimate was above unity, and in 2 of these, the lower limit of the 95% CI of the risk ratio was above unity, giving a significant increased risk. For 6 sites, the point estimate was below unity, but in none was the upper limit of the 95% CI below unity, so there was not strong evidence for an increased risk.

**Figure 1:**
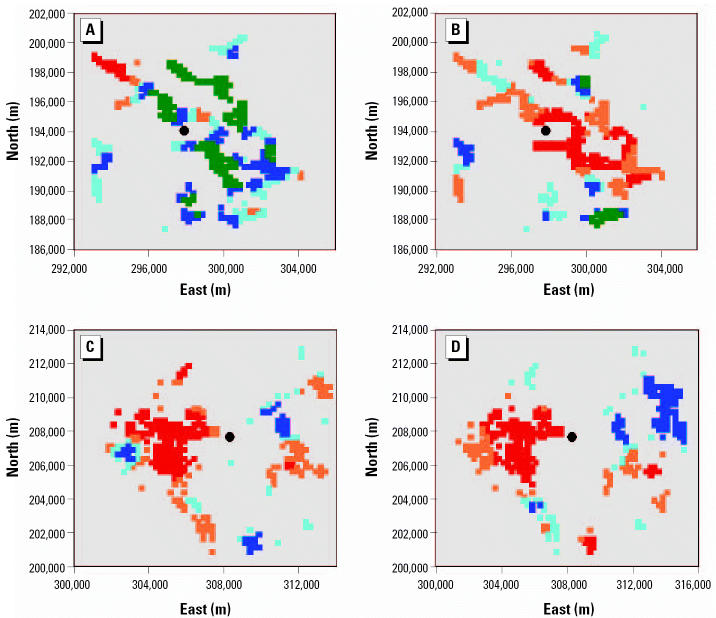
Spatial distribution of smoothed standardized (observed–expected/expected) risk ratios for congenital anomalies within squares with 250-m sides in two areas. Colored squares are quintiles of the ratio observed–expected/square root of expected in descending order: red, orange, light blue, dark blue, green. (A) Nantygwyddon before opening; (B) Nantygwyddon after opening; (C) Trecatti before opening; (D) Trecatti after opening.

For illustrative purposes the distribution of smoothed risk estimates around two sites, Nantygwyddon and Trecatti, is shown in Figure 1. The population distribution followed the contours of the valleys. In the first case, an area of increased relative risk became apparent in the period after the site opened immediately adjacent to the site. In the second case, areas of increased relative risk were apparent before and after opening in the same location to the west of the landfill.

### 1998–2000.

There were 97,292 births identified on the Child Health System, and CARIS identified 2,633 congenital anomalies in live births, of which 2,534 (2.6%) were matched to these births. No data from this system were available for the periods before the landfills opened. Consequently, only the relation between risk of congenital anomalies and distance from the landfills after opening could be studied. Overall, within 2 km of the 20 landfills studied for 1998–2000, the standardized risk ratio was 1.04 (95% CI, 0.88–1.21) (Table 2). For the sites licensed to take chemicals, the standardized risk ratio was 1.08 (95% CI, 0.75–1.41). For those introducing containment, it was 1.19 (95% CI, 0.90–1.48), and for those that introduced gas control, it was 1.14 (95% CI, 0.88–1.41). These results include the 7 sites where both containment and gas control were introduced. When these 7 sites were excluded from analysis, the standardized risk ratio for containment only was 1.19 (95% CI, 0.90–1.48); for gas control only, the numbers were insufficient to allow an accurate calculation of a CI. If the definition of exposure is changed to living within 3 km, as for the pre-1997 data, the standardized risk ratio for containment only was 1.04 (95% CI, 0.72–1.36) and for gas control only was 1.77 (95% CI, 1.20–2.34).

## Discussion

We tested the null hypothesis that the opening of landfill sites taking mainly domestic and commercial waste was not associated with an increased risk of congenital anomalies in nearby residents, although we did not have sufficient statistical power to explore relationships with subgroups of congenital anomalies with much precision. Data on actual exposures to chemicals that might be emitted from the landfills are not available within the United Kingdom. We have now found, when summing over all the landfills, that the rates of all congenital anomalies identified through statutory notifiable data increased significantly after sites opened until 1997, and this was the case whether we took 2- or 3-km distances as the definition of exposure. We were concerned to find out if this increase persisted after 1997 using more recent complete and accurate data collected, but we did not find an increased risk over the 3 years from 1998 through 2000.

Several other landfill studies have been carried out with varying findings (Barry and Bove 1997; Croen et al. 1997; Dolk et al. 1998; Elliott et al. 2001; Geschwind et al. 1992; Kharrazi et al. 1997; Marshall et al. 1997; Orr et al. 2002; Vrijheid et al. 2002a). The most recent study in the United States by Orr et al. (2002) was a case–control study of birth defects in racial or ethnic minority children born to mothers in California. They found an increased risk for all defects of 1.12 (95% CI, 0.98–1.27) in births to mothers living in the same census tract as a hazardous waste site. The EUROHAZCON study also looked at 21 landfill sites in Britain, France, Italy, Denmark, and Belgium that took hazardous waste in a case–control study of nonchromosomal (Dolk et al. 1998) and chromosomal (Vrijheid et al. 2002a) congenital anomalies. Living within 3 km was associated with a 33% increased risk compared with living 3–7 km away. Risk decreased with distance from the sites, and adjustment for area socioeconomic level did not remove the effect. Elliott et al. (2001) considered all landfill sites in Great Britain, most of which were small and held only domestic waste. They found that 80% of the British population lived within 2 km of such sites. There was a small excess risk when examining sites only after they opened, but a secondary analysis comparing before and after opening on about half the sites did not find an excess risk. However, their control population was essentially rural, and therefore, differences between the study and control groups could possibly account for these findings. Also, bias of differential reporting of congenital anomalies by hospitals may have occurred. In our study there was a 3-fold variation in rates around different hospitals, and this was considered in the analysis.

There are a number of well-recognized problems in interpreting ecologic studies of point sources of pollution (Vrijheid 2000). One potential source of bias is the use of surrogate measures of exposure in the absence of individual-level exposures. The proxy exposure measures of distance from site assumes that the toxic effect is airborne because smell is often the provoking event, but contamination of ground-water and percolation through soil must also be considered (Vrijheid et al. 2002b). Distance of residence from the landfill cannot take into account duration of residence or the proportion of time that individuals spent away from their residence. Nor is it possible to detect directional patterns using concentric circles. The radii of 2 km chosen by Elliott et al. (2001) were pragmatic, maximizing the power of comparisons while remaining within plausible estimates of the range of chemicals dispersed from a site; no sound evidence has yet been published to measure human exposures with distance from land-fills in the United Kingdom, but expert opinion suggests that small particles from landfills may be detectable up to 3 km away (WHO 2001). We are exploring alternatives to using concentric circles (James et al. 2003). In the two identified sites presented in this article, the distribution of increased risk is not uniform with distance, and the patterns are quite different. Pooling results across sites will mask these differences.

Another problem in studying single sites is that most have resident populations too small to give sufficient power to demonstrate a small but important public health effect. Aggregation of data around several sites is therefore necessary to obtain statistical power, but the chemical content of different sites may vary enormously and dilute any real associations with specific pollutants. Also, the influence of those sites with larger populations will predominate. It is also necessary to use broad groups of conditions to obtain sufficient numbers of cases; specific congenital anomalies may have different etiologies that cannot be identified in pooled studies (Elliott and Wakefield 2000). Data on what was disposed of in the landfills are not available, nor is there insufficient information about what chemicals come from specific sites to enable analyses of sensible subgroups of sites.

We have shown that the rate of congenital anomalies in residents living within 2 km of landfills was significantly higher after landfills opened, but these data cannot prove or disprove a causal link. The existence of other sources of pollution in the same temporal and geographic relationship to the population as the landfill sites is not a likely explanation. An alternative hypothesis is that opening of landfills triggered a change in the makeup of the population living nearby or triggered changes in social and other lifestyle or medical care characteristics of mothers living there (e.g., uptake of screening and terminations for congenital anomalies), although we have no data to suggest this happened. A single snapshot of area deprivation would not adjust for this. Furthermore, area-based deprivation measures may not accurately reflect socioeconomic status of the individual, and we did not have data on maternal smoking, nutrition, or other lifestyle factors that may confound the relationship between residence near landfills and congenital anomalies.

The increased rates of congenital anomalies around landfill sites in Wales persisted until 1998. In the next 2 years, the rate ratios fell to close to 1. Is this simply due to less biased surveillance data, or has improved management of sites in recent years reduced exposure of the population to hazardous chemicals? Data quality of the congenital anomalies registers used up to 1998 is certainly an important issue because considerable underreporting is well documented. However, even if our adjustment for reporting hospitals did not fully compensate for possible differential reporting by distance of landfills, such bias would not explain the changes in rates with distance after opening of the landfills. Furthermore, such a fall in risk is not readily explained by the alternative hypothesis of social change. Intriguingly, using data up to 1997 in those sites that introduced gas control, which is likely to reduce exposure of the public to emissions, some time after opening, showed a fall in the risk ratio. In those sites where movement of landfill material would have increased because of the introduction of new containment cells, possibly temporarily increasing emissions, there was an increased risk ratio. This pattern was not evident in the enhanced data after 1998, although numbers of cases were very small.

## Conclusions

In Wales, pooling data from populations living within 2 km of 24 landfill sites that opened from 1983 through 1997, we have found that the ratio of observed to expected rates of congenital anomalies increased by about 40%. This increase did not persist in data collected from 1998 through 2000. Causal inference is hampered by lack of data on individual-level exposures. Other socioeconomic and lifestyle factors should be considered in the etiology of higher area rates of congenital anomalies, but there is also an urgent need to characterize and minimize exposure of individuals (particularly pregnant women) to environmental chemicals that may be emitted from any industrial site of concern. Continued enhanced surveillance is also needed to overcome the bias for differential ascertainment of congenital anomalies.

## Figures and Tables

**Table 1:** Births, congenital anomalies, and standardized risk ratios (95% CIs) within 2 km of the landfill sites, 1983–1997.

	Before			After			
Sites	Congenital anomalies	Total births	Standardized risk ratio	Congenital anomalies	Total births	Standardized risk ratio	Ratio after:before
Before opening and after opening of sites	182	14,230	0.87 (0.75–1.00)	173	15,451	1.21 (1.04–1.40)	1.39 (1.21–1.72)
As above but including four sites with significant expansion	223	16,948	0.89 (0.78–1.01)	202	17,860	1.22 (1.06–1.39)	1.38 (1.13–1.67)
Fifteen sites introducing containment after opening	12	766	1.11 (0.64–1.81)	102	8,097	1.27 (1.05–1.53)	1.15 (0.63–2.29)
Eight sites with containment but no gas control	3	380	0.79 (0.29–1.90)	28	2,757	1.16 (0.81–1.63)	1.47 (0.45–7.56)
Ten sites introducing gas control after opening	64	3,617	1.39 (1.09–1.75)	20	2,310	1.03 (0.67–1.52)	0.74 (0.42–1.24)
Three sites with gas control but no containment	1	113	—	0	88	—	—
Six sites that accept chemical waste	38	2,358	1.15 (0.84–1.55)	43	4,319	1.25 (0.93–1.65)	1.08 (0.68–1.72)

—, Numbers are too small to quote reliable CIs.

**Table 2:** Births, congenital anomalies, and standardized risk ratios (95% CIs) within 2 km of the landfill sites, 1998–2000.

Sites	Congenital anomalies	Total births	Standardized risk ratio
Before opening and after opening of sites	178	5,297	1.04 (0.88–1.21)
As above but including four sites with significant expansion	204	6,134	1.04 (0.88–1.21)
Fifteen sites introducing containment after opening	124	3,827	1.11 (0.92–1.31)
Eight sites with containment but no gas control	70	2,129	1.19 (0.90–1.48)
Ten sites introducing gas control after opening	55	1,756	1.14 (0.88–1.41)
Three sites with gas control but no containment	1	58	1.03^a^
Six sites that accept chemical waste	42	1,216	1.08 (0.75–1.41)

a Numbers are too small to quote reliable CIs.
